# Therapeutic efficacy of superficial vein-anastomosed spiral propeller flap in elderly patients with foot and ankle soft tissue defects

**DOI:** 10.3389/fsurg.2025.1629340

**Published:** 2025-07-18

**Authors:** Guangmou Chen, Zhidong Liao, Guoqiang Ruan, Huirun Chen, Guihe Lv, Jiashang Cai, Jin Chen, Bo Liang

**Affiliations:** ^1^ Department of Orthopaedics, Affiliated Hospital of Guangdong Medical University, Zhanjiang, China; ^2^ Department of Orthopaedics, Zhanjiang Central Hospital, Guangdong Medical University, Zhanjiang, China

**Keywords:** spiral propeller flap, superficial vein anastomoses, soft tissue defects, elderly, foot and ankle

## Abstract

**Background:**

This study aimed to evaluate the efficacy of superficial vein anastomoses spiral propeller flaps in improving venous return and reducing postoperative complications for elderly patients with foot and ankle soft tissue defects.

**Methods:**

A prospective study involving 15 elderly patients (65–90 years) underwent spiral propeller flap repair between 2020 and 2021. Flap perfusion, swelling, venous patency (Doppler ultrasound), and ankle function (AOFAS score) were assessed.

**Results:**

All flaps survived, with 2 cases of distal superficial necrosis healing after dressing. Postoperative swelling improved from Grade II (*n* = 10) to Grade I (*n* = 13) at 6 months. AOFAS scores showed 86.7% excellent/good outcomes.

**Conclusion:**

Venous anastomosis in spiral propeller flaps significantly enhances flap survival and functional recovery in elderly patients with comorbidities.

## 1 Introduction

Addressing defects covering one-third of the distal leg and Achilles tendon area poses a challenge for both orthopedic and plastic surgeons. Although free flaps have been widely used by plastic surgeons for these defects, they are time-consuming (1, 2). Local fasciocutaneous flaps can reconstruct moderate to mild defects, but their availability and mobility are limited. These flaps have limited availability and mobility in the distal leg (3).

In recent years, with advancements in the anatomical knowledge of perforator vessels along the lower limb axis, the methods for reconstructing lower limb soft tissue defects have evolved. In recent decades, spiral propeller flaps with perforator vessels have become increasingly popular. The concept of the spiral propeller flap was first described by Hyakusosku et al. in 1991 (4). In 2011, the Tokyo Consensus defined the spiral propeller flap as a “perforator flap with two skin islands.”

The spiral propeller flap consists of two skin paddles, one larger and one smaller, separated by a nutrient perforator vessel corresponding to the pivot point (5). The flaps are designed in the shape of an asymmetrical airplane propeller and can rotate 180° around a pivot point to cover adjacent defects. The spiral propeller design offers safe, reliable, and technically straightforward coverage, with a very low donor site morbidity rate (6–8).

Additionally, this method has a low failure rate, low risk of secondary surgery, and allows preservation of the blood supply to the foot and ankle (9). The distal leg perforator flap is a commonly used method for repairing soft tissue defects in the foot and ankle, but complications are more likely in elderly patients due to poor tissue perfusion and venous outflow obstruction, leading to flap necrosis (10–12).

This study aimed to repair soft tissue defects in the elderly foot and ankle using superficial vein anastomoses spiral propeller flaps, describing our surgical technique, evaluating clinical outcomes, and determining complications in patients with soft tissue defects in the distal leg area.The propeller flap is an established concept, and this study contributes by expanding the evaluation across a larger patient population, more detailed clinical outcome analysis, and standardized surgical techniques. In comparison with prior case reports and small-scale studies, this work offers a more extensive assessment of superficial vein-anastomosed propeller flaps in elderly patients.

## 2 Materials and methods

### 2.1 General information

From January 2020 to December 2021, 15 cases of elderly patients with skin and soft tissue defects at the foot and ankle were treated at Guangdong Medical University Affiliated Hospital and Guangdong Medical University Affiliated Zhanjiang Center People's Hospital. The study group comprised 15 patients, including 6 males and 9 females, aged 65–90 years. Defect locations were as follows: 3 cases at the anterior ankle, 5 cases at the medial malleolus, and 7 cases at the posterior ankle and heel. Causes of defects included trauma (9 cases), diabetic foot (3 cases), and infection (3 cases). Coexisting conditions included hypertension (5 cases), diabetic foot (3 cases), fractures or osteomyelitis (8 cases), coronary heart disease (1 case), and chronic obstructive pulmonary disease (2 cases).

### 2.2 Preoperative preparation

Preoperative treatment was tailored to relevant diseases. Patients with hypertension received antihypertensive medication, and diabetic patients had their fasting blood glucose controlled to below 8 mmol/L. Patients with chronic obstructive pulmonary disease received internal medicine treatment. Infected wounds in the ankle area were debrided, dressed, or treated with vacuum sealing drainage (VSD) and antibiotics until stabilized.

### 2.3 Surgical procedure

Flaps with pedicles were designed following the principles of “point, line, area, and arc.” The perforator vessel location in the flap was determined preoperatively using Doppler ultrasound and marked. A spiral propeller design was centered around the point where the vessel pierced the skin. The pivot point was the perforator vessel, with the larger paddle covering the wound and the smaller paddle covering the donor area. The procedure included: (1) Flap Harvest: Thorough debridement and hemostasis were achieved in the recipient site. A tourniquet was applied at the proximal thigh to maintain a bloodless field during flap dissection, which was released after flap dissection to assess perfusion. The distal end of the flap was dissected first, the perforator vessel was located, and the perforator vessel was preserved within the flap. The flap's position was readjusted around the pivot point of the perforator vessel. The distal part of the flap was designed as the smaller paddle, and the proximal part as the larger paddle. The distal part of the flap was dissected from the deep fascia to the proximal end, preserving the perforator vessel within the flap. Surrounding fibrous tissues and fascia around the perforator vessel were removed to ensure that the pedicle of the perforator vessel remained lax. Adequate blood supply was observed to the flap, with the flap connected to the perforator vessel and surrounding fascia, forming two paddle-like ends. (2) Selection of Superficial Vein Branch: Under microscopic magnification (×10–20), one or two superficial veins were preserved within the flap during the dissection of the proximal end. A matching-diameter superficial vein was selected from the recipient site, with the proximal end used for anastomosis. The veins at both the donor site and the corresponding recipient site were meticulously dissected to obtain adequate length for tension-free anastomosis. (3) Wound Coverage: Depending on the condition of the flap and pedicle, the flap was rotated 120–180 degrees clockwise or counterclockwise to ensure that the perforator vessel was not twisted or compressed. The larger paddle covered the wound, and the smaller paddle covered the donor area of the flap. The remaining skin was sutured, and if the remaining flap was not sutured, skin grafting was performed. Microsurgical venous anastomosis was performed between the superficial vein of the flap and the recipient-site superficial vein under high-power magnification (×20–25). After applying microvascular clamps to both ends, the veins were meticulously coapted using interrupted 10-0 nylon sutures, with patency confirmed by the milking test and observed spontaneous flow. After wound closure, a rubber sheet drain was placed beneath the flap for postoperative drainage.

### 2.4 Postoperative evaluation

Flaps were monitored hourly for the first 24 h postoperatively, then every 4 h until postoperative day 10 to assess perfusion, capillary refill, and signs of congestion. ∼∼Flap perfusion was closely monitored up to 10 days after surgery. ∼∼ Bedrest was strictly maintained for 3 days to minimize pedicle tension, followed by progressive dangling exercises (initially 5 min increments, 3 times daily) to promote venous adaptation. Sutures were removed at 2 weeks postoperatively, followed by ankle joint and limb function exercises after 3 weeks. (1) Flap Swelling Assessment: Flap swelling was evaluated using a 4-grade scale. The extent of swelling was assessed at early (7 days) and later stages (6 months) after surgery: Grade I: Slight flap swelling (−); Grade II: Flap swelling with preserved skin lines (+); Grade III: Marked flap swelling with absent skin lines (++); Grade IV: High flap swelling with tension blisters (+++). (2) Assessment of Venous Anastomosis Patency: Venous anastomosis patency was evaluated using Doppler ultrasound one month after surgery. (3) Flap Appearance Satisfaction Survey: A questionnaire survey was conducted 6 months postoperatively to assess patient satisfaction with the appearance of the flap. (4) Assessment of Ankle Joint Function: Ankle joint and hindfoot function were assessed 6 months postoperatively using the American Orthopaedic Foot & Ankle Society (AOFAS) ankle and hindfoot scoring system (13).

## 3 Results

Fifteen cases of wound repair using superficial vein anastomoses spiral propeller flaps were performed (Table 1). Among them, 5 cases used the peroneal artery perforator flap, 3 cases used the lateral malleolus perforator flap, 3 cases used the posterior tibial artery perforator flap, and 4 cases used the medial malleolus perforator flap. The flap dimensions ranged from 6.0 cm × 3.5 cm to 20 cm × 5.5 cm. Fourteen flaps survived entirely, while one flap exhibited a small area of superficial necrosis at the distal end, approximately 2.5 cm × 1.5 cm in size. This necrotic area healed after dressing changes over four weeks. Incision healing was excellent, and all skin grafts survived. At six months postoperatively, ankle joint function recovered well in 13 cases, while 2 cases experienced limited mobility (Table 2).

**Table 1:** Patient's basic medical record information.

Case number	Age/sex	Etiology	The site of the defect	Concomitant disease
1	70/F	Traumatic defect	Posterior ankle and heel	Diabetes
2	66/M	Traumatic defect	medial malleolus	Hypertension and chronic obstructive pulmonary disease
3	67/M	Infection defect	Posterior ankle and heel	hypertension
4	68/F	Traumatic defect	Posterior ankle and heel	Fracture or Osteomyelitis
5	66/M	Traumatic defect	medial malleolus	Fracture or Osteomyelitis
6	83/F	Traumatic defect	Anterior ankle	Fracture or Osteomyelitis
7	90/M	diabetic foot	medial malleolus	Diabetes and Hypertension and coronary heart disease
8	65/F	Traumatic defect	Anterior ankle	Fracture or Osteomyelitis
9	77/F	Traumatic defect	Posterior ankle and heel	hypertension
10	72/F	Infection defect	Posterior ankle and heel	None
11	68/M	Traumatic defect	Posterior ankle and heel	Fracture or Osteomyelitis
12	66/F	Traumatic defect	medial malleolus	Fracture or Osteomyelitis
13	68/M	diabetic foot	medial malleolus	hypertension and chronic obstructive pulmonary disease
14	82/F	diabetic foot	Posterior ankle and heel	diabetes
15	70/F	Traumatic defect	Anterior ankle	Fracture or Osteomyelitis

F, female; M, male.

**Table 2:** Detailed flap data and reconstruction results.

Case number	Vascular source	Venous source	Flap dimensions	Arc of rotation (degrees)	Donor site closure	Complications	Skin flap swelling		AOFAS ankle and hind foot score
							7 d	3 m	
1	Posterior tibial artery	Small saphenous vein	7 × 3.5 cm (15 cm^2^)	180°	Primary closure	None	Ⅰ°	Ⅰ°	Excellent
2	Peroneal artery	Small saphenous vein	12 × 6 cm (72 cm^2^)	120°	Skin Graf	None	Ⅱ°	Ⅰ°	Excellent
3	Peroneal artery	Dorsalis pedis vein	17 × 6 cm (102 cm^2^)	180°	Skin Graf	None	Ⅰ°	Ⅰ°	Good
4	Peroneal artery	Small saphenous vein	9 × 5 cm (45 cm^2^)	170°	Primary closure	None	Ⅰ°	Ⅰ°	Excellent
5	Posterior tibial Artery + Peroneal artery	Branch of great saphenous vein	20 × 5.5 cm (110 cm^2^)	180°	Skin Graf	None	Ⅰ°	Ⅰ°	Excellent
6	Peroneal artery	Small saphenous vein	11 × 3.5 cm (38.5 cm^2^)	170°	Skin Graf	Partial superficial necrosis	Ⅱ°	Ⅰ°	Good
7	Posterior tibial Artery + Peroneal artery	Branch of great saphenous vein	7 × 4 cm (28 cm^2^)	180°	Primary closure	None	Ⅱ°	Ⅰ°	Fair
8	Posterior tibial artery	Branch of great saphenous vein	9 × 3 cm (27 cm^2^)	170°	Skin Graf	None	Ⅰ°	Ⅰ°	Good
9	Posterior tibial artery	Branch of great saphenous vein	14 × 5.5 cm (77 cm^2^)	180°	Skin Graf	None	Ⅰ°	Ⅰ°	Fair
10	Peroneal artery	Small saphenous vein	3 × 3.5 cm (32 cm^2^)	170°	Primary closure	None	Ⅱ°	Ⅱ°	Excellent
11	Posterior tibial Artery + Peroneal artery	Small saphenous vein	8 × 3.5 cm (28 cm^2^)	180°	Primary closure	None	Ⅱ°	Ⅱ°	Excellent
12	Peroneal artery	Small saphenous vein	15 × 5 cm (75 cm^2^)	180°	Skin Graf	None	Ⅱ°	Ⅰ°	Excellent
13	Posterior tibial artery	Branch of great saphenous vein	8 × 4.5 cm (36 cm^2^)	180°	Primary closure	None	Ⅱ°	Ⅰ°	Good
14	Posterior tibial Artery + Peroneal artery	Branch of great saphenous vein	11 × 4 cm (44 cm^2^)	180°	Skin Graf	None	Ⅱ°	Ⅰ°	Good
15	Posterior tibial Artery + Peroneal artery	Branch of great saphenous vein	12 × 4.5 cm (54 cm^2^)	180°		None	Ⅱ°	Ⅰ°	Excellent

F, female; M, male.

Flap Swelling Assessment: At 7 days postoperatively, there were 6 cases of Grade I swelling and 9 cases of Grade II swelling. At 6 months postoperatively, there were 13 cases of Grade I swelling and 2 cases of Grade II swelling.

Venous Anastomosis Patency: One month after surgery, Doppler ultrasound confirmed that the venous anastomosis was patent in all 15 cases.

Flap Appearance Satisfaction Survey: All 15 cases were followed up for 12 months postoperatively, and a questionnaire survey conducted at 6 months revealed that all patients were satisfied with the appearance of the flap.

Assessment of Ankle Joint Function: At 6 months postoperatively, according to the AOFAS ankle and hindfoot scoring system, there were 8 cases rated as excellent, 5 cases as good, and 2 cases as fair, with an excellent and good rate of 86.7%.

### 3.1 Typical case 1

**Figure 1:**
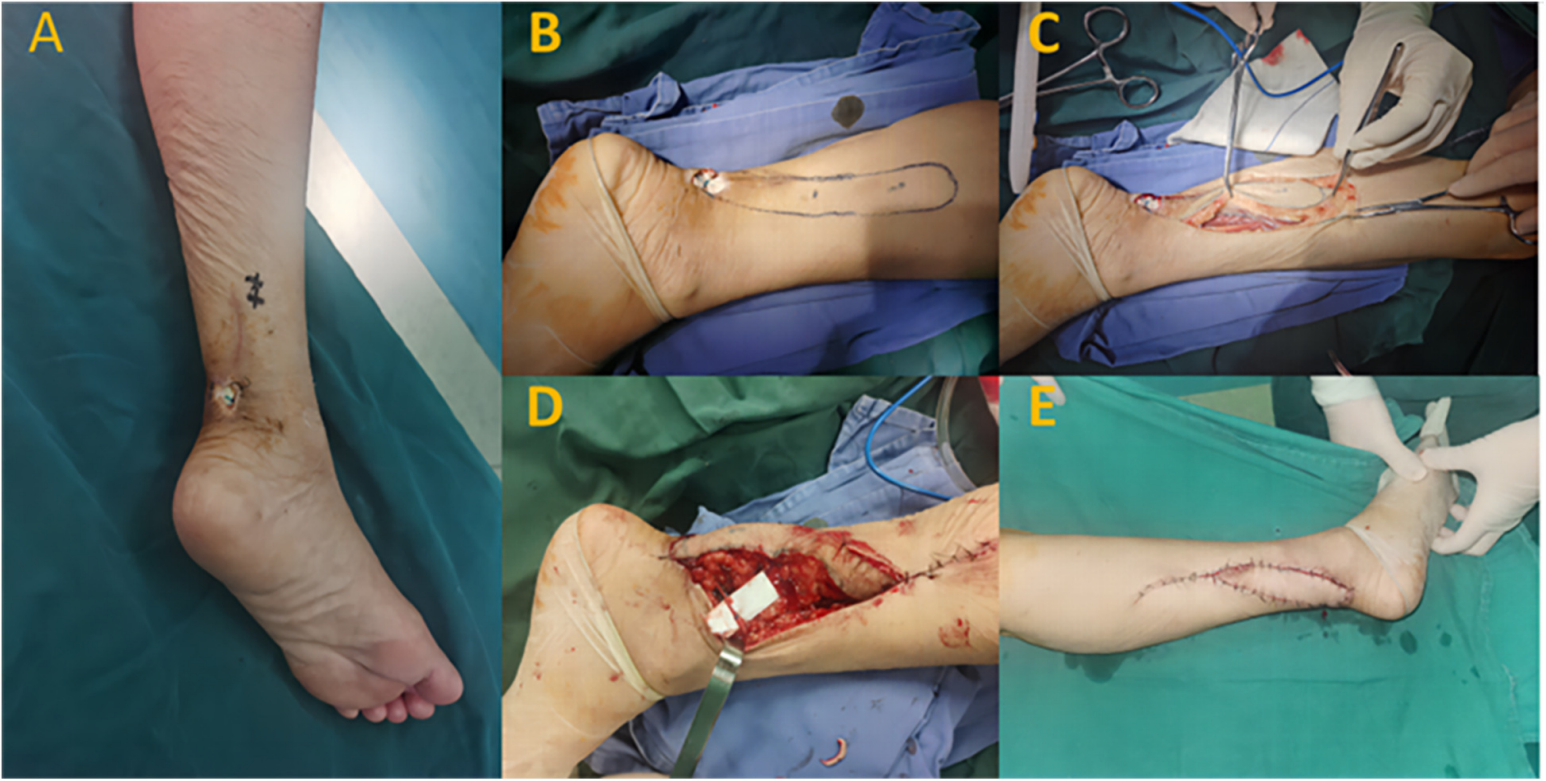
Typical case 1. **(A)** Posterior heel skin defect exposing the Achilles tendon. (B) Intraoperative planning of the superficial vein anastomosed propeller flap. **(C)** Dissection of the flap with preservation of the superficial vein. **(D)** Flap transfer and anastomosis. **(E)** Postoperative view showing good blood supply.

A 70-year-old female patient with a history of left Achilles tendon rupture surgery presented with wound dehiscence exposing the Achilles tendon. She also had type 2 diabetes, for which she irregularly took oral antidiabetic medication. After admission, routine examinations were conducted, wound secretions were cultured for bacteria, wound dressings were enhanced, and blood glucose control was actively managed. After 5 days of hospitalization with stable patient and wound conditions, surgery was performed to repair the wound using a superficial vein anastomoses spiral propeller flap from the posterior tibial artery. Preoperatively, Doppler ultrasound was used to locate the perforator vessel at the distal end of the posterior tibial artery (located 10 cm above the medial malleolus, along the posterior edge of the tibia). Following debridement, the wound size was approximately 3.5 cm × 3.0 cm. The flap area was designed to be approximately 7.0 cm × 3.5 cm, centered around the perforator vessel at the distal end of the posterior tibial artery. During flap dissection, an appropriate length of superficial vein was preserved within the flap. The pedicled flap was rotated clockwise to cover the wound, and the superficial vein of the flap was end-to-end anastomosed to a small tributary vein in the recipient area. At 10 days postoperatively, the flap was completely viable, with good blood supply and no significant swelling (Grade I swelling). At 6 months postoperatively, the flap had healed well, exhibited satisfactory appearance, good texture, an average sensation discrimination of 12 mm, and an AOFAS ankle function score of 95 points (Figure 1).

### 3.2 Typical case 2

A 66-year-old male patient suffered a right tibia fracture due to trauma, resulting in a soft tissue defect with exposed bone. The patient also had hypertension and chronic obstructive pulmonary disease (COPD). Upon admission, routine assessments were performed. In the initial phase of hospitalization, the patient underwent fracture reduction and external fixation along with VSD treatment. After a two-week hospital stay and stabilization of the wound, the patient underwent a surgery involving anastomosis of the superficial vein for a fibular artery perforator spiral propeller flap. The remaining wound in the donor site of the flap was repaired through skin grafting.

**Figure 2:**
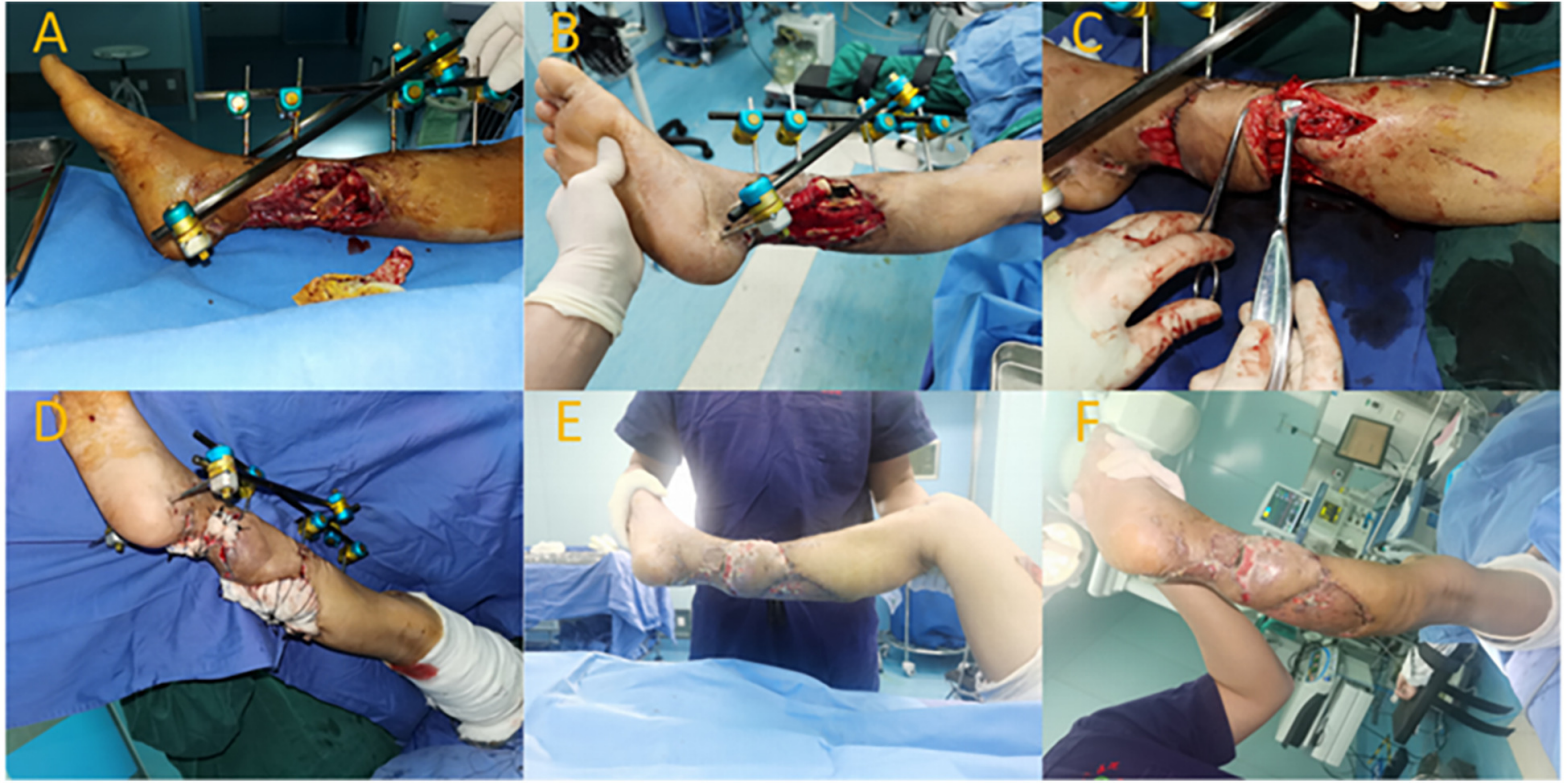
Typical case 2. **(A,B)** Soft tissue defect on the posterior aspect of the right lower leg after trauma, with exposed tibia. **(C)** Intraoperative rotation and anastomosis of the perforator flap's superficial vein. **(D)** Transfer of the flap to cover the wound; skin graft used for donor site. **(E,F)** Postoperative view showing survival of the flap and skin graft.

Preoperatively, ultrasonography was used to locate the perforator vessel at the distal end of the fibular artery (located approximately 15 cm above the lateral malleolus). Following debridement, the wound measured about 6 cm × 8 cm. The flap area was designed to be approximately 6 cm × 12 cm. The rotation point for the flap was chosen at the perforator site of the posterior fibular artery. During flap dissection, an appropriate length of superficial vein was preserved within the flap. The pedicled flap was rotated clockwise to cover the wound, and the superficial vein within the flap was end-to-side anastomosed with a branch of the recipient's lesser saphenous vein. Ten days postoperatively, the flap had fully survived with a healthy blood supply, showing no significant swelling. The degree of swelling was assessed as level II. Six months after the surgery, the flap had healed well with satisfactory appearance and texture. The patient's ankle function was evaluated using the AOFAS score, and it achieved a score of 93, indicating good functional recovery of the right ankle (Figure 2).

### 3.3 Typical case 3

**Figure 3:**
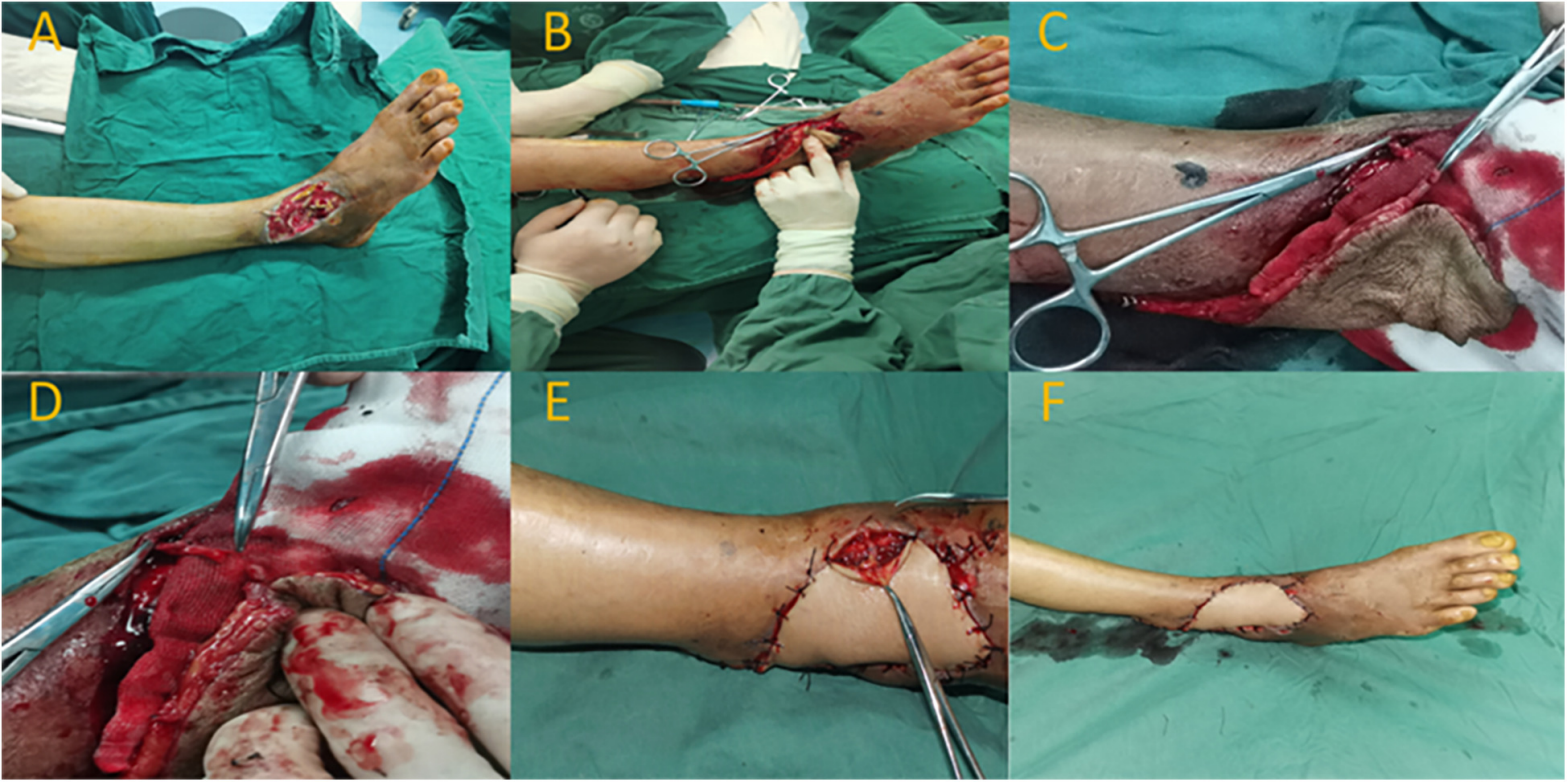
Typical case 3. **(A)** Soft tissue defect on the anterior aspect of the right ankle with tendon exposure. **(B)** Intraoperative flap rotation to cover the wound. **(C,D)** Anastomosis of superficial veins. **(E)** Venous anastomosis site. **(F)** Postoperative flap with good blood supply.

A 67-year-old male patient presented with a soft tissue defect on the dorsal aspect of the right foot due to infection. After admission, the patient received antimicrobial treatment. In the first-stage surgery following admission, wound debridement and VSD therapy were performed. After a week of hospitalization and stabilization of the wound condition, the patient underwent a repair procedure using an anastomosed superficial venous perforator flap from the posterior tibial artery. Preoperatively, ultrasound Doppler was used to locate the perforating vessel at the distal end of the posterior tibial artery (located approximately 7.0 cm above the lateral malleolus). Following wound debridement, the wound measured around 5.0 cm × 7.0 cm. The flap was designed with an approximate size of 6.0 cm × 17.0 cm. The perforator point of the posterior tibial artery was chosen as the rotation point. During flap elevation, an appropriate length of superficial vein was preserved within the flap. The pedicle flap was then rotated clockwise to cover the wound area. The superficial vein of the flap was anastomosed end-to-end with a branch vessel of the dorsal vein of the foot. At 10 days postoperatively, the flap exhibited complete viability, with good blood supply and no significant swelling. The degree of swelling was assessed as grade I. Six months after the surgery, the flap showed satisfactory healing, with pleasing appearance and texture. The patient achieved an AOFAS ankle score of 85, indicating good functional recovery of the right ankle (Figure 3).

## 4 Discussion

### 4.1 Selection of repair methods for soft tissue defects in the ankle region of elderly patients

There are various methods for repairing soft tissue defects in the ankle region. These include the sural nerve neurovascular flap, local perforator flap, free flap, and cross-leg flap, which are all conventional repair methods (14–16). The free flap technique requires microsurgical vascular anastomosis and is more complex, while the cross-leg flap requires strict immobilization of the limb after surgery, resulting in long ischemia times and patient discomfort (17, 18). Many elderly patients have underlying conditions such as hypertension and diabetes, which can lead to poor overall health and an inability to tolerate complex and lengthy surgeries. The sural nerve nutrient vascular flap relies solely on nerve nutrient blood vessels for blood supply, but many elderly patients also have hypertension and diabetes, which can cause peripheral vascular disease, leading to insufficient blood supply and flap necrosis. The vascular perforator flap has a stable vascular pedicle, a reliable flap blood supply system, multiple perforators, and doesn't require complex microsurgical techniques. After rotation, the propeller flap may exhibit a relatively favorable contour, which could potentially reduce the need for secondary surgical revisions (19).

### 4.2 Venous outflow issues with pedicled perforator flaps

Pedicled perforator flaps have a smaller pedicle tissue volume and greater flexibility for rotation. However, as the tissue volume decreases and rotation angle increases, there is an increased risk of complications due to venous outflow obstruction, especially in distally based pedicled perforator flaps (20). According to the modified Starling-Landis model, five major factors affect tissue fluid transudation, including capillary forces pushing fluid out (capillary hydrostatic pressure + interstitial colloidal osmotic pressure) and forces pulling fluid into capillaries (interstitial fluid pressure + plasma colloid osmotic pressure), with lymphatic pressure compensating for the difference between them. After flap harvesting, these five factors change, leading to increased pressure pushing fluid out of capillaries compared to fluid entering, resulting in tissue edema and flap swelling. Improved venous return in the flap is achieved gradually through neovascularization, but severe venous outflow obstruction can cause flap congestion, tension blisters, and even thrombosis. When blood circulation is below 100 g of skin tissue with a minimum oxygen consumption of 2l/min, irreversible necrosis can occur in ischemic parts of the flap (21).

### 4.3 Research on hemodynamics of distally based perforator flaps

Multi-area perforator flaps consist of multiple perforating sections, with different types of vascular anastomoses in the vascular stream bed and intervascular shunts, greatly affecting flap hemodynamics. To ensure the survival of multi-area perforator flaps, arterial hyper-perfusion and venous superdrainage are commonly used in clinical practice. However, there is still no consensus on the optimal dimensions and distance from the perforator that can be safely designed without the need for supercharge or additional vein anastomosis. The distribution of intradermal vessels, number of perforating sections, and types of vascular anastomosis are key factors that may influence the flap's perfusion and overall outcome (22). In practical clinical work, when a single perforating vessel nourishes an entire multi-area perforator flap, poor viability in the distal tissue often occurs, even leading to necrosis. To enhance distal flap survival, “hyper-perfusion” of arterial anastomoses and “superdrainage” of venous anastomoses can be performed on the distal flap, providing better reliability, versatility, and safety for venous anastomosed perforator flaps (23).

The position, diameter, axiality of perforating vessels, number of perforating bodies, and anastomosis types are all critical factors influencing the hemodynamics of multi-area perforator flaps, crucial for flap survival. Both flap blood perfusion and outflow can be quantified, enabling precise preoperative flap design and guiding the choice between hyper-perfusion and superdrainage during flap harvesting. Based on current research on multi-area perforator flaps, when harvesting an extended multi-area perforator flap, it's suggested to consider carrying a distal perforating artery or vein for hyper-perfusion and superdrainage during surgery, with a preference for hyper-perfusion. A distal perforating vessel occlusion test can be used during surgery to assess distal blood circulation. If severe venous congestion occurs, superdrainage should be employed, and if there is poor or absent dermal bleeding, hyper perfusion is needed (22).

### 4.4 Superdrainage to improve venous outflow in propeller flaps

Superdrainage involves individually anastomosing the vein of a pedicled flap after flap transfer (24). Chang and others conducted relevant studies using a perforator flap rat model in 2004 and 2007, showing that venous super drainage significantly improved microcirculation at the distal end of the flap. The positioning of the super drainage was found to be more effective at the distal end of the flap compared to the proximal end. Studies have indicated that the spiral pedicled perforator flap with great saphenous vein anastomosis can effectively cover small to moderate defects, reduce venous congestion, and lower overall complications (25–27). When anastomosing superficial veins of the flap, the choice of recipient vein is crucial. The vein at the distal end of the flap should be selected to maximize drainage, while the vein in the recipient area should be located near the proximal end of the limb (venous return side) to allow venous blood from the flap to flow towards the heart (drainage function), rather than near the distal end of the limb (venous inflow side), which would direct limb venous blood into the flap (perfusion function) and exacerbate flap swelling.

### 4.5 Discussion of surgical indications, contraindications, and precautions

The purpose of this surgical approach is to improve venous outflow in the flap, enhance flap survival, and utilize the principle of flap superdrainage, which holds significant clinical value. Indications for the technique include: (1) Improving venous outflow in pedicled perforator flaps (with or against the pedicle direction), especially when perforating vessels are small, pedicle tissue volume is limited, and the rotation angle is large. (2) Managing venous crises in flaps after surgery to rescue flaps with venous outflow obstruction. Contraindications include: (1) Poor recipient skin conditions, such as severe scarred or infected wounds. (2) Poor recipient vascular conditions, such as severe diabetes-related vascular diseases or other vascular disorders. (3) Absence of suitable venous trunks in the flap area for anastomosis with recipient veins. During surgery, attention should be given to: (1) Preoperative detection and localization of the perforator's anatomical position using Doppler ultrasound. If the anatomical position is too high or affects adequate flap rotation, or if the perforating vessel is small and flap rotation affects blood supply, alternative methods should be considered. (2) Thorough consideration of flap pedicle adjustment during surgical design to ensure the pedicle is tension-free after flap transfer. During grafting and compression in the supply area, care should be taken to avoid compressing the pedicle and obstructing blood supply and return. When increasing the size of the flap, it is recommended to lengthen the incision rather than forcefully pulling the flap, as excessive tension can compromise flap blood circulation. Interrupted sutures at the edge of the flap should have larger distances between stitches, and knot tying should not be too tight. Small distal veins can be left unligated to facilitate blood return. (3) Tension-free anastomosis of superficial veins with recipient veins, ensuring that the anastomosis site is well-covered with tissue and avoiding excessive tension during skin closure, which could compress the venous anastomosis site. Although imaging modalities such as MRI and ultrasound have been utilized in previous studies to quantify flap volume and perfusion, our study relied on clinical scoring systems and Doppler evaluation due to the retrospective design and imaging resource constraints. We acknowledge this as a limitation and propose incorporating quantitative imaging tools in future prospective investigations to enhance the objectivity of data analysis.

## 5 Conclusion

The spiral pedicled flap is a reliable choice for reconstructing small to moderate lower limb defects, yielding excellent clinical outcomes and a low incidence of donor site morbidity. Elderly patients often have poor tissue perfusion, and venous outflow obstruction can lead to flap necrosis or partial necrosis. Anastomosing superficial veins in the spiral pedicled flap effectively improves venous outflow, reduces postoperative swelling, enhances flap survival in elderly patients, and results in satisfactory repairs with excellent clinical outcomes and minimal donor site morbidity. The spiral pedicled flap with venous anastomosis offers better clinical results compared to other flaps.

## Data Availability

The raw data supporting the conclusions of this article will be made available by the authors, without undue reservation.
